# Corner-Point and Foreground-Area IoU Loss: Better Localization of Small Objects in Bounding Box Regression

**DOI:** 10.3390/s23104961

**Published:** 2023-05-22

**Authors:** Delong Cai, Zhaoyun Zhang, Zhi Zhang

**Affiliations:** 1 School of Electrical Engineering and Intelligentization, DongGuan University of Technology, Dongguan 523000, Chinazhangz@dgut.edu.cn (Z.Z.); 2 School of Computer Science and Technology, DongGuan University of Technology, Dongguan 523000, China

**Keywords:** object detection, loss function, small object, bounding box regression

## Abstract

Bounding box regression is a crucial step in object detection, directly affecting the localization performance of the detected objects. Especially in small object detection, an excellent bounding box regression loss can significantly alleviate the problem of missing small objects. However, there are two major problems with the broad Intersection over Union (IoU) losses, also known as Broad IoU losses (BIoU losses) in bounding box regression: (i) BIoU losses cannot provide more effective fitting information for predicted boxes as they approach the target box, resulting in slow convergence and inaccurate regression results; (ii) most localization loss functions do not fully utilize the spatial information of the target, namely the target’s foreground area, during the fitting process. Therefore, this paper proposes the Corner-point and Foreground-area IoU loss (CFIoU loss) function by delving into the potential for bounding box regression losses to overcome these issues. First, we use the normalized corner point distance between the two boxes instead of the normalized center-point distance used in the BIoU losses, which effectively suppresses the problem of BIoU losses degrading to IoU loss when the two boxes are close. Second, we add adaptive target information to the loss function to provide richer target information to optimize the bounding box regression process, especially for small object detection. Finally, we conducted simulation experiments on bounding box regression to validate our hypothesis. At the same time, we conducted quantitative comparisons of the current mainstream BIoU losses and our proposed CFIoU loss on the small object public datasets VisDrone2019 and SODA-D using the latest anchor-based YOLOv5 and anchor-free YOLOv8 object detection algorithms. The experimental results demonstrate that YOLOv5s (+3.12% Recall, +2.73% mAP@0.5, and +1.91% mAP@0.5:0.95) and YOLOv8s (+1.72% Recall and +0.60% mAP@0.5), both incorporating the CFIoU loss, achieved the highest performance improvement on the VisDrone2019 test set. Similarly, YOLOv5s (+6% Recall, +13.08% mAP@0.5, and +14.29% mAP@0.5:0.95) and YOLOv8s (+3.36% Recall, +3.66% mAP@0.5, and +4.05% mAP@0.5:0.95), both incorporating the CFIoU loss, also achieved the highest performance improvement on the SODA-D test set. These results indicate the effectiveness and superiority of the CFIoU loss in small object detection. Additionally, we conducted comparative experiments by fusing the CFIoU loss and the BIoU loss with the SSD algorithm, which is not proficient in small object detection. The experimental results demonstrate that the SSD algorithm incorporating the CFIoU loss achieved the highest improvement in the AP (+5.59%) and AP75 (+5.37%) metrics, indicating that the CFIoU loss can also improve the performance of algorithms that are not proficient in small object detection.

## 1. Introduction

Object detection is an important task in computer vision. Currently, object detection algorithms can be mainly classified into two types: anchor-based object detection algorithms, such as Faster R-CNN [1], SSD [2], and YOLOv5 [3], and anchor-free object detection algorithms, such as CornerNet [4], FCOS [5], and YOLOv8 [6]. In these different object detection frameworks, bounding box regression is a crucial step in predicting rectangular boxes for locating objects. Generally, the regression performance of bounding boxes reflects the localization ability of the object detection algorithm. Therefore, a suitable loss function plays a vital role in improving the performance of bounding box regression. Currently, there are two types of loss functions for bounding box regression:

- Loss based on the ${𝓁}_{n}$-norm. The literature [1,7,8,9,10] calculates the ${𝓁}_{n}$-norm distance between the corresponding coordinate points of two boxes to measure the distance between the predicted box and the target box. However, the literature [11] argues that the ${𝓁}_{n}$-norm loss only considers the difference between two boxes and ignores their spatial relationship and containment relationship, thus proposing another loss based on Intersection over Union (IoU) between two boxes to measure the actual regression performance of the predicted box and the target box.
- Loss based on Broad IoU. The IoU loss function only considers the difference between the predicted box and the target box, without taking into account the intersection and anchor information between the two boxes. When the predicted box and the target box do not overlap, IoU cannot reflect the distance between them, and its corresponding loss function cannot calculate gradients, making it impossible to optimize the parameters of the predicted box in the next step. To address this issue, many researchers have proposed IoU-based loss functions that incorporate spatial information errors between the predicted box and the target box into the original IoU loss function, which can better improve the accurate positioning ability of the predicted box. These loss functions include the original IoU loss and various improved versions, which are collectively referred to as broad IoU losses. Due to their excellent performance in measuring the actual differences between two bounding boxes, BIoU losses have been widely used in object detection algorithms. Currently, the mainstream BIoU losses include GIoU loss [12], DIoU and CIoU loss [13], and EIoU loss [14]. Therefore, the BIoU losses can be defined as Equation (1)
  (1) $${𝓛}_{BIoU}\left(B,B^{gt}\right)=1-BIoU.$$

BIoU can be defined by Equation (2):

(2) $$BIoU=\frac{\left|B\cap B^{gt}\right|}{\left|B\cup B^{gt}\right|}-R\left(B,B^{gt}\right),$$

where B and $B^{gt}$ represent the predicted box and the target box, respectively. $R\left(B,B^{gt}\right)$ represents the penalty term, which is mainly used to accelerate model convergence or to better fit the predicted box to the target box.

**Figure 1:**
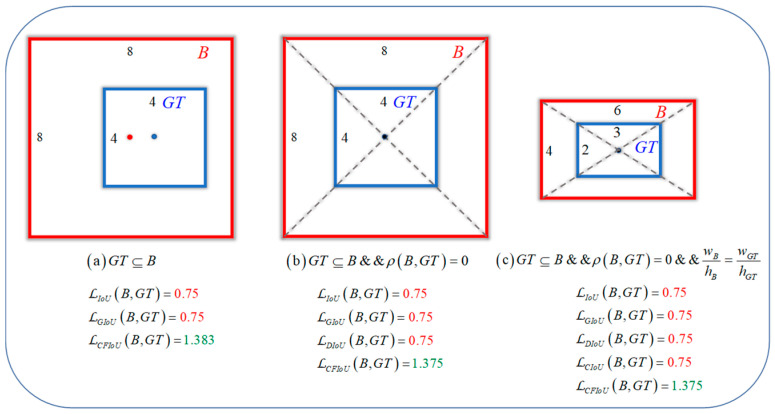
Three position relationships between the predicted box B and the target box GT during the regression process, along with their corresponding loss values for bounding box regression. (**a**–**c**) represent the three position relationships between the predicted box and the target box. The red value represents the BIoU loss value degenerating to the IoU loss value when the predicted box approaches the target box. The green value represents the CFIoU loss value. By comparing the results, it can be seen that CFIoU loss does not suffer from the same degeneration issue as BIoU loss.

**Figure 2:**
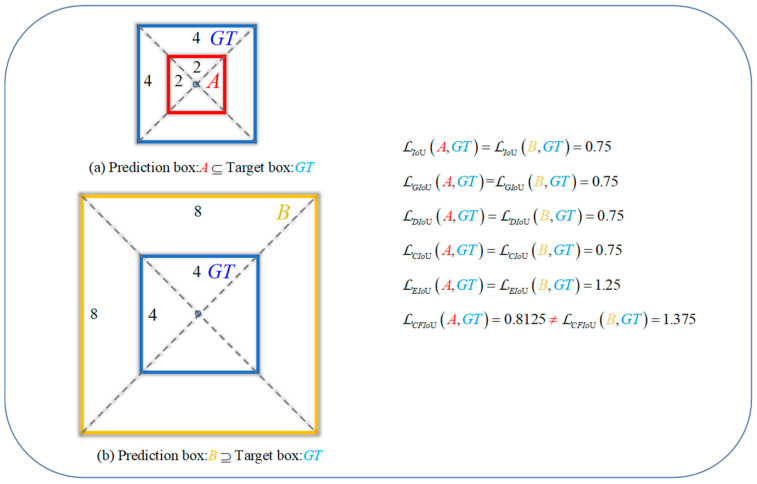
Left: The relative positions of predicted boxes A and B with respect to the target box GT. Right: The values of BIoU losses and CFIoU loss in cases (**a**,**b**). In (**a**), predicted box A is inside the target box GT, while in (**b**), predicted box B is outside the target box GT. The fitting effect of predicted box A is better than that of predicted box B. Therefore, the loss function should impose a greater penalty on predicted box B to improve its regression performance. Unfortunately, BIoU losses cannot distinguish the regression situation of the predicted boxes in (**a**,**b**), as their loss values are equal in these two cases. In contrast, the CFIoU loss results in a larger loss value for predicted box B, which is better.

These BIoU loss functions improve the localization ability of the predicted boxes by considering the discrepancies between the center point distances, overlap areas, and width-height information of both boxes. However, as the predicted box gradually approaches the target box, the GIoU loss, DIoU loss, and CIoU loss directly degrade to the IoU loss. This can be seen as the situation in Figure 1, where BIoU losses start to degrade to IoU loss when the predicted box and target box geometric center points are close or overlap. It should be noted that IoU loss cannot reflect the intersection and anchor box information of the two boxes, so it cannot offer a more effective means of fitting for the predicted box. On the other hand, in the process of gradually fitting the predicted box to the target box, BIoU losses do not take into account the spatial information of the true target, which leads to the localization loss function being unable to know the true fitting degree between the predicted box and target box, and thus unable to provide more accurate information for the next optimization of the predicted box. This can be seen as the situation in Figure 2, where for the two different fitting situations (i.e., different relative positions and overlap situations) of boxes A and B, BIoU losses yield identical values for both predicted boxes. In actuality, the regression performance of the two predicted boxes is completely different, and it can be considered that the regression situation of box A is better than that of box B, so box B should be punished more (i.e., loss value) to obtain a faster and better fitting effect. In small object detection, this approach of disregarding real target information is detrimental to the localization effect of small objects and can easily lead to the missed detection of small objects.

In this paper, we propose a corner-point and foreground-area IoU loss (CFIoU loss). We add a penalty term for deviation in corner distances to IoU loss, which provides more position parameter update information and solves the problem of BIoU losses degrading to IoU loss when the predicted box and target box geometric center points are close or overlap. In addition, we also use the spatial information of the target, i.e., the target foreground region, as part of the loss function to provide more accurate fitting information to predicted boxes with poorer regression effects, enabling them to fit better to the target box. This design solves the problem of BIoU losses being unable to distinguish between predicted boxes with different fitting situations during bounding box regression.

To evaluate the effectiveness of our proposed method, we conducted simulation experiments on bounding box regression. In addition, to evaluate the performance of the CFIoU loss, we integrated it into the latest anchor-based YOLOv5 and anchor-free YOLOv8 object detection algorithms, and quantitatively compared BIoU losses and CFIoU loss on the VisDrone2019 [15] and SODA-D [16] small object public datasets.

The contributions of this paper are summarized as follows:

1. CFIoU loss is a loss function designed for bounding box regression, which provides faster and better regression performance than BIoU losses.
2. To address the deficiency of BIoU losses when the predicted box approaches the target box, we propose a loss term based on the corner point distance deviation.
3. To utilize target information in bounding box regression optimization, we propose an adaptive loss term. This approach is particularly effective for small targets with limited information.
4. Our proposed method can be easily integrated into existing anchor-based and anchor-free object detection algorithms to achieve improved performance on small targets.

## 2. Related Works

This section provides a brief overview of the related work, which includes the problems associated with BIoU losses and the strategies that utilize target foreground information.

### 2.1. BIoU Losses for Bounding Box Regression

#### 2.1.1. Limitations of IoU Loss

IoU loss [11], which stands for Intersection over Union loss, was originally introduced in face detection as a comprehensive measure that takes into account all relevant attributes. Equation (3) defines this loss function:

(3) $${𝓛}_{IoU}=1-IoU.$$

IoU can be unified as Equation (4):

(4) $$IoU=\frac{\left|B\cap B^{gt}\right|}{\left|B\cup B^{gt}\right|},$$

where B and $B^{gt}$ represent the predicted box and the target box, respectively, and they can be of arbitrary size. IoU is scale-invariant, meaning that the spatial scale of the predicted box B and target box $B^{gt}$ does not affect their similarity measurement. However, IoU loss has two major drawbacks: (1) if two bounding boxes have no intersection, IoU loss will always be 1, failing to reflect their actual distance; (2) IoU cannot distinguish between different alignments of the two bounding boxes, making it impossible to identify the better alignment.

#### 2.1.2. Limitations of Generalized IoU Loss (GIoU Loss)

The GIoU loss [12] was proposed to address the shortcomings of the original IoU loss. Its definition is shown in Equation (5):

(5) $${𝓛}_{GIoU}=1-IoU+\frac{\left|C-B\cup B^{gt}\right|}{\left|C\right|},$$

where C represents the minimum closed bounding box that encompasses both B and $B^{gt}$. Compared to the IoU loss, the GIoU loss has superior dynamic behavior and can reflect the spatial position between two bounding boxes even when IoU = 0, as illustrated in Figure 1a. However, the GIoU loss still has some limitations. For instance, when there is a containment relationship between two bounding boxes, the GIoU loss will degenerate into the IoU loss and cannot differentiate the relative position of the two boxes. Additionally, when there is a significant vertical direction error between the two boxes, the GIoU loss becomes unstable and can lead to difficulties in loss convergence.

#### 2.1.3. Limitations of Distance IoU Loss (DIoU Loss)

DIoU loss [13] adds a penalty term for the distance between the centers of two boxes based on IoU loss, accelerating the convergence process of the model. It can be defined by Equation (6):

(6) $${𝓛}_{DIoU}=1-IoU+\frac{\rho^{2}\left(b,b^{gt}\right)}{{\left(l^{C}\right)}^{2}},$$

where b and $b^{gt}$ represent the center points of the predicted box B and target box $B^{gt}$ respectively. $\rho \left(b,b^{gt}\right)={\left\|b-b^{gt}\right\|}_{2}$ represents the Euclidean distance between the two points, and $l^{C}$ represents the diagonal distance of the minimum closed bounding box C that contains both B and $B^{gt}$. DIoU loss mitigates the slow convergence problem of GIoU loss to some extent. However, DIoU loss still cannot describe the overlap information between the two boxes well. Moreover, when the center points of the two boxes coincide completely, both GIoU and DIoU losses will still degenerate into IoU loss, as shown in Figure 1b.

#### 2.1.4. Limitations of Complete IoU Loss (CIoU Loss)

CIoU loss [13] is an improved version of DIoU loss that incorporates aspect ratio information of two bounding boxes. It can better reflect the distance and alignment between the two boxes, thus further improving the quality and speed of regression. It can be defined by Equation (7):

(7) $${𝓛}_{CIoU}=1-IoU+\frac{\rho^{2}\left(b,b^{gt}\right)}{{\left(l^{C}\right)}^{2}}+\alpha v,$$

where $v=\frac{4}{\pi^{2}}{\left(\arctan\frac{w^{gt}}{h^{gt}}-\arctan\frac{w}{h}\right)}^{2}$ and $\alpha =\frac{v}{\left(1-IoU\right)+v}$ are used to represent the differences in aspect ratios between the two boxes. However, the aspect ratios used in CIoU loss are relative values, which have a certain degree of uncertainty. When the two boxes are aligned in the diagonal direction, as shown in Figure 1c, the GIoU, DIoU, and CIoU losses degenerate directly into the IoU loss.

#### 2.1.5. Limitations of Efficient IoU Loss (EIoU Loss)

EIoU loss [14] is an improvement over CIoU loss. It uses the difference in width and height between the target and predicted bounding boxes instead of aspect ratio information. This approach enables more direct minimization of the distance between the target and predicted boxes, improving the convergence speed and accuracy of object localization. EIoU loss can be defined using Equation (8):

(8) $${𝓛}_{EIoU}=1-IoU+\frac{\rho^{2}\left(b,b^{gt}\right)}{{\left(l^{C}\right)}^{2}}+\frac{\rho^{2}\left(w,w^{gt}\right)}{{\left(w^{C}\right)}^{2}}+\frac{\rho^{2}\left(h,h^{gt}\right)}{{\left(h^{C}\right)}^{2}},$$

where $w^{C}$ and $h^{C}$ represent the width and height of the minimum closed bounding box C that contains both B and $B^{gt}$. Although the EIoU loss function performs better than the previous IoU loss function, neither the EIoU nor the IoU loss utilizes the foreground region information of the true object, making it difficult to determine the relative position and matching degree of the predicted and target boxes. Therefore, models trained using BIoU losses may perform poorly in detecting small objects.

### 2.2. Strategies for Utilizing Object Foreground Information

Class imbalance is a classic problem in object detection tasks, and the imbalance between foreground and background samples is a subproblem. Anchor-based object detection algorithms have a significant impact on addressing the foreground–background sample imbalance problem. However, due to the sparsity of object boxes and the IoU (Intersection over Union) matching strategy between boxes, negative samples have a larger contribution than positive samples in minimizing the loss, leading to a bias toward the negative sample class in model performance.

Therefore, various methods for enhancing foreground information utilization have been proposed at different stages of object detection. For example, the spatial attention module (SAM) [17] enhances features of the foreground region from the feature map obtained from the region proposal network (RPN) [1]. PISA (Prime Sample Attention) [18] proposes an importance-based sample selection method, IoU-HLR (Hierarchical Local Rank), which weights predicted boxes based on the IoU value between the predicted box and the ground truth box. Predicted boxes with richer foreground information are assigned greater weight. Another method proposed in the literature [19] is an IoU-balanced sampling technique to extract more training samples from challenging cases. VarifocalNet [20] uses ATSS (Adaptive Training Sample Selection) [21] to define foreground points in a framework without anchors. Designing a balanced foreground–background loss function in the classification stage is also a common method. For instance, AP loss (Average Precision loss) [22] reweights examples, DR loss (Distributional Ranking loss) [23] reweights the distribution of background examples based on the distribution of foreground examples, and focal loss [24] recalibrates the weight distribution between positive and negative examples. It is worth noting that the utilization of target foreground region information is relatively low in the box regression stage compared to other stages.

In conclusion, this article proposes the following hypotheses. First, using a border distance metric as a replacement for the traditional geometric center distance in object detection can effectively avoid the problem of the BIoU losses degenerating into the IoU loss when the distance between two bounding boxes is close. This results in an acceleration of the model’s convergence speed and an improvement in its fitting performance. Second, adding a penalty term aimed at the foreground region of the object to the bounding box regression loss function can enable the localization loss function to fully consider the relative position and overlap information of two bounding boxes. This, in turn, enables the correction of poorly regressed prediction boxes by imposing a stronger penalty, thereby enhancing the model’s ability to locate small objects.

## 3. Methods

Based on the above conjecture, we have designed a new loss function for bounding box regression. In this section, we introduce the methodology of this loss function and its corresponding advantages. Generally, the IoU-based loss can be defined as Equation (9), which is derived from Equations (1) and (2):

(9) $$𝓛=1-IoU+R\left(B,B^{gt}\right),$$

where $R\left(B,B^{gt}\right)$ represents the penalty term between the predicted box B and the target box $B^{\mathrm{gt}}$. To resolve the aforementioned issue, we designed a suitable penalty term, which can be defined as Equation (10):

(10) $$R\left(B,B^{gt}\right)=R_{Cor}\left(B,B^{gt}\right)+R_{Fore}\left(B,B^{gt}\right).$$

### 3.1. Penalty Term $R_{Cor}\left(B,B^{gt}\right)$ Based on Corner Point Distance

$R_{Cor}\left(B,B^{gt}\right)$ represents the penalty term for the distance between the corresponding corners of two bounding boxes, which can be defined as Equation (11):

(11) $$R_{Cor}\left(B,B^{gt}\right)=\frac{\sum_{i=1}^{4}\rho^{2}\left(x_{i},x_{i}^{gt}\right)}{4\times {\left(l^{C}\right)}^{2}},$$

where $\rho \left(x_{i},x_{i}^{gt}\right)$ denotes the Euclidean distance between the $i-\mathrm{th}\left(i=1,2,3,4\right)$ pair of corners of the predicted box B and the target box $B^{\mathrm{gt}}$. $R_{Cor}\left(B,B^{gt}\right)$ can effectively avoid the degradation of BIoU loss to IoU loss when the distance between two boxes is close (as shown in Figure 1). According to theoretical derivation, when the center points of the predicted and target boxes coincide, the loss term based on center point distance cannot provide further optimization help. However, the loss function based on the corner point distance can continue to work effectively until the predicted and target boxes completely overlap, assuming $P_{1}=\sum_{i=1}^{4}\rho^{2}\left(x_{i},x_{i}^{gt}\right)$, $P_{2}=\rho^{2}\left(b,b^{gt}\right)$. The gradient of $P_{1}$ with respect to the center point and scale parameters $\left(x,y,w,h\right)$ of the predicted box is defined by Equation (11a).

(11a) $$\begin{array}{l} \frac{\partial P_{1}}{\partial x}=8\times \left(x-x^{gt}\right),\;\\ \frac{\partial P_{1}}{\partial y}=8\times \left(y-y^{gt}\right),\\ \frac{\partial P_{1}}{\partial w}=2\times \left(w-w^{gt}\right),\;\\ \frac{\partial P_{1}}{\partial h}=2\times \left(h-h^{gt}\right). \end{array},$$

where $\left(x^{gt},y^{gt},w^{gt},h^{gt}\right)$ represent the center point and scale parameters of the target box. The gradient of $P_{2}$ with respect to the predicted box parameters $\left(x,y,w,h\right)$ is defined by Equation (11b).

(11b) $$\begin{array}{l} \frac{\partial P_{2}}{\partial x}=2\times \left(x-x^{gt}\right),\\ \frac{\partial P_{2}}{\partial y}=2\times \left(y-y^{gt}\right),\\ \frac{\partial P_{2}}{\partial w}=0,\\ \frac{\partial P_{2}}{\partial h}=0. \end{array},$$

By Equations (11a) and (11b), it can be observed that during gradient updating of the predicted box parameters $\left(x,y,w,h\right)$, the distance information $P_{1}$ of the four corner points can provide gradient information for both the center point parameter $\left(x,y\right)$ and the scale parameter $\left(w,h\right)$ of the predicted box. However, the distance information $P_{2}$ between the center points can only provide gradient information for the center point parameter $\left(x,y\right)$ of the predicted box.

### 3.2. Penalty Term $R_{Fore}\left(B,B^{gt}\right)$ Based on Target Foreground Information

To address the issue of BIoU losses lacking the utilization of target foreground information, we propose a penalty term $R_{Fore}\left(B,B^{gt}\right)$ to represent the target foreground information. We believe that an ideal loss function for bounding box localization should be able to fully utilize the foreground information of the actual target. Specifically, for small objects, as they contain less information, they are more likely to be missed. $R_{Fore}\left(B,B^{gt}\right)$ can be defined by Equation (12):

(12) $$R_{Fore}\left(B,B^{gt}\right)=\left(1-\mu \right)\frac{{\left|B-B^{gt}\right|}^{2}}{{\left|C\right|}^{2}}+\mu \frac{{\left|C-B^{gt}\right|}^{2}}{{\left|C\right|}^{2}},\;\mu =\left\{\begin{array}{ccc} 0 & if & \rho \left(b,b^{gt}\right)\neq 0\\ 1 & if & \rho \left(b,b^{gt}\right)=0 \end{array}\right..$$

Here, μ is used to determine whether the geometric centers of the predicted box and the target box coincide.

- When the geometric centers of the predicted and target bounding boxes do not coincide, we use the method of directly minimizing the size difference between the predicted box and the real target foreground to speed up the regression of the predicted box.
- When the geometric centers of the predicted and target bounding boxes coincide, we use the difference between the minimum enclosing region C and the real target foreground region to distinguish the contributions of the foreground and background regions in the penalty function. If the proportion of the foreground region in the minimum enclosing region C of the predicted and target bounding boxes is small (the proportion of the background region is large), it indicates that the regression effect of the current predicted box is not good, so the predicted box needs to be punished more severely. In particular, for small objects, which have limited foreground information and are prone to be missed, using the foreground information in the minimum enclosing region C can help their bounding boxes obtain more advantageous gradient information for regression.

### 3.3. Corner-Point and Foreground-Area IoU Loss

Based on Equations (11) and (12), a novel bounding box loss function, namely the CFIoU loss, can be defined by Equation (13):

(13) $${𝓛}_{CFIoU}=1-IoU+R\left(B,B^{gt}\right)=1-IoU+\frac{\sum_{i=1}^{4}\rho^{2}\left(x_{i},x_{i}^{gt}\right)}{4\times {\left(l^{C}\right)}^{2}}+\left[\left(1-\mu \right)\frac{{\left|B-B^{gt}\right|}^{2}}{{\left|C\right|}^{2}}+\mu \frac{{\left|C-B^{gt}\right|}^{2}}{{\left|C\right|}^{2}}\right],\mu =\left\{\begin{array}{ccc} 0 & if & \rho \left(b,b^{gt}\right)\neq 0\\ 1 & if & \rho \left(b,b^{gt}\right)=0 \end{array}\right.$$

**Figure 3:**
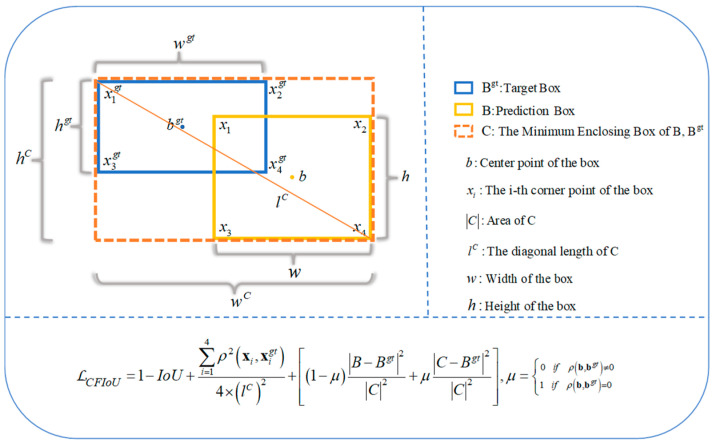
CFIoU Loss for Bounding Box Regression.

The specific definitions of the parameters and their variables are referenced in Figure 3.

**Figure 4:**
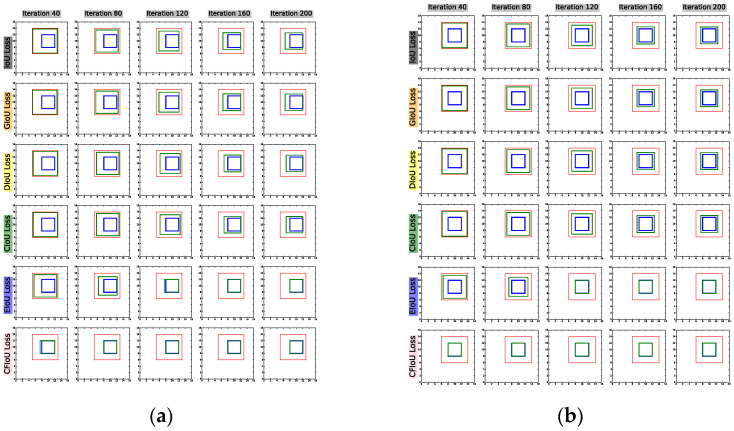
Visualization of the bounding box regression process. (**a**): The process where the predicted box fits to the target box in Figure 1a. (**b**): The process where the predicted box fits to the target box in Figure 1b. The red box represents the initial position of the predicted box, the blue box represents the position of the target box, and the green box represents the predicted box during the fitting process.

**Figure 5:**
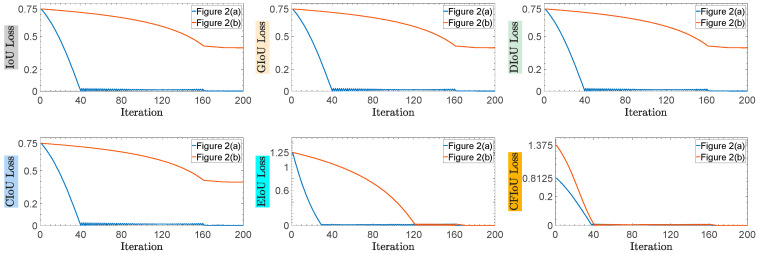
The process of minimizing various losses during the fitting of the two types of predicted boxes in Figure 2a,b to the target box.

We compare the CFIoU loss with the BIoU losses and draw the following conclusions:

- The proposed CFIoU loss inherits some properties of the BIoU loss:

(1) The CFIoU loss is still scale-invariant for regression problems.

(2) The CFIoU loss can provide the direction of movement for the predicted box when it does not overlap with the target box. Additionally, it still follows the characteristics of DIoU, CIoU, and EIoU losses based on the normalized distance between two bounding boxes to accelerate the regression of the predicted box.

(3) When the predicted box completely matches the target box, ${𝓛}_{CFIoU}={𝓛}_{IoU}={𝓛}_{GIoU}={𝓛}_{DIoU}={𝓛}_{CIoU}={𝓛}_{EIoU}=0$. When the distance between the two boxes is far apart, ${𝓛}_{CFIoU}={𝓛}_{GIoU}={𝓛}_{DIoU}={𝓛}_{CIoU}={𝓛}_{EIoU}\to 2$.

- The CFIoU loss has the following advantages over the BIoU losses:

(1) The CFIoU loss can effectively solve the problem of the BIoU losses degenerating into the IoU loss when the predicted box overlaps with or approximates the geometric center of the target box;

(2) The CFIoU loss makes full use of the foreground area information of the target box, allowing it to differentiate between predicted boxes with different regression effects and to give greater penalties to boxes with poorer regression effects, helping them to better fit the target box. This is lacking in the BIoU losses.

- To better illustrate the superiority of the CFIoU loss over BIoU losses, we designed a visualization process for bounding box regression based on the positions of the predicted and target boxes in Figure 1 and Figure 2.

(1) We start with the predicted and target box positions in Figure 1a,b, and use BIoU and CFIoU losses as regression losses for the predicted box. We minimize this loss using gradient descent over 200 iterations to obtain the final regression results, as shown in Figure 4. We observed that the CIoU, DIoU, and GIoU losses degrade to the IoU loss, causing the predicted box to fail to fit the target box completely. In contrast, the EIoU and CFIoU losses avoid this degradation issue and enable the predicted box to fit the target box completely. Among these two loss functions, the CFIoU loss converges much faster than the EIoU loss due to its richer gradient information. Additionally, the simulation results in Figure 4b are similar to those in Figure 4a, indicating that the CFIoU loss can avoid the degradation issue and achieve faster convergence and better fitting performance for the predicted box.

(2) We conducted regression based on the predicted and target box positions in Figure 2a,b, using the BIoU and CFIoU losses as regression losses. We minimized the losses using gradient descent and obtained the final regression results after 200 iterations. Specifically, we visualized the process of minimizing various loss functions during the predicted box fitting to the target box in Figure 5. Through the visualization results in Figure 5, we found that for the two different regression results of the predicted boxes in Figure 2a,b, the BIoU loss values were equal: ${𝓛}_{IoU}\left(a\right)={𝓛}_{IoU}\left(b\right)=0.75$, ${𝓛}_{GIoU}\left(a\right)={𝓛}_{GIoU}\left(b\right)=0.75$, ${𝓛}_{DIoU}\left(a\right)={𝓛}_{DIoU}\left(b\right)=0.75$, $\;{𝓛}_{CIoU}\left(a\right)={𝓛}_{CIoU}\left(b\right)=0.75$, ${𝓛}_{EIoU}\left(a\right)={𝓛}_{EIoU}\left(b\right)=1.25$. This suggests that both the poorly and well-regressed predicted boxes received the same penalty, which caused the poorly regressed predicted boxes to lack additional gradient information to better or more quickly fit. In the IoU loss, GIoU loss, DIoU loss, and CIoU loss graphs in Figure 5, we found that the poorly regressed predicted box in Figure 2b could not fit the target box, while in the EIoU loss graph, although the poorly regressed predicted box in Figure 2b could eventually fit the target box, it did so much slower than the well-regressed predicted box in Figure 2a. In the CFIoU loss graph, the poorly regressed predicted box in Figure 2b and the well-regressed predicted box in Figure 2a received different penalties: ${𝓛}_{CFIoU}\left(a\right)=0.8125\neq {𝓛}_{CFIoU}\left(b\right)=1.375$. Clearly, the poorly regressed predicted box in Figure 2b received more penalties than the well-regressed predicted box in Figure 2a, which is due to the addition of information about the target foreground area in the CFIoU loss. From the CFIoU loss graph, it can be seen that the poorly regressed predicted box in Figure 2b achieved faster fitting speed (the losses of the predicted box and the target box started to converge after 40 iterations) while maintaining a good fitting effect.

## 4. Experiments and Discussion

### 4.1. Datasets and Evaluation Metrics

**Figure 6:**
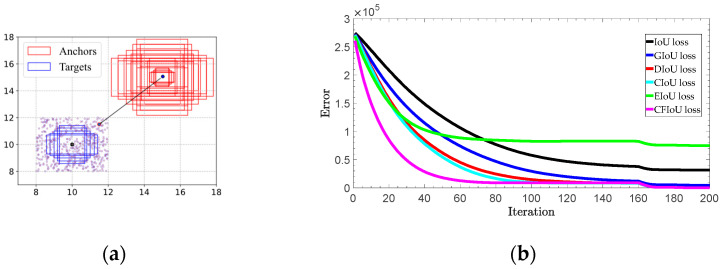
Simulation experiment for bounding box regression: (**a**) 62,500 regression cases were used by considering different distances, scales and aspect ratios; (**b**) total regression error.

We conducted experiments on both synthetic and real datasets. To investigate the advantages of the CFIoU loss, we designed a simulation experiment covering various aspects of small object bounding boxes, such as distance, scale, and aspect ratio. Specifically, we selected five target boxes with different aspect ratios (i.e., 1:2, 2:3, 1:1, 3:2, and 2:1) as the ground truth boxes, all with an area of four. We fixed the center points of these five boxes at coordinates (10, 10) and randomly placed 500 anchor boxes at each point in a rectangular region with a length and width of 4. These anchor boxes include cases with and without overlap with the ground truth boxes. At each point, we set five different scales (i.e., 1, 4, 8, 12, and 16) and five aspect ratios (i.e., 1:2, 2:3, 1:1, 3:2, and 2:1), resulting in a total of 12,500 = 500 × 5 × 5 anchor boxes, as shown in Figure 6a. All of these anchor boxes should perfectly fit the five ground truth boxes of different sizes. Therefore, we obtained a total of 62,500 = 5 × 5 × 5 × 500 regression cases.

Regarding the real datasets, we chose the VisDrone2019 and SODA-D datasets for small object detection and tracking experiments. We used the VisDrone2019-DET-train and SODA-D train datasets to train the model and the VisDrone2019-DET-val and SODA-D val datasets to supervise the training process to avoid overfitting. Finally, we tested the model’s performance on the VisDrone2019-DET-test and SODA-D test datasets and reported our ablation study on the VisDrone2019-DET-test dataset. We selected the YOLO format evaluation metrics, mean average precision (mAP) and recall (R), as the main evaluation metrics. It is worth noting that all our experiments were conducted on the following hardware devices: (1) Intel(R) Core(TM) i5-6500 CPU @ 3.20GHz; (2) NVIDIA GeForce RTX 3090 and NVIDIA GeForce RTX 1080Ti.

### 4.2. Simulation Experiment

To validate the effectiveness of the CFIoU loss on a large number of anchor box regressions, we designed a simulation experiment using a synthetic dataset (Figure 6a) to simulate the regression of bounding boxes. By specifying a loss function 𝓛, we can simulate the regression process of the bounding box under each situation using the gradient descent method. The current prediction for the predicted box $B_{n,s}$ after t iterations can be obtained using Equation (14):

(14) $$B_{n,s}^{t}=B_{n,s}^{t-1}+\eta \nabla B_{n,s}^{t-1},$$

where $B_{n,s}^{t}$ is the predicted box after t iterations, $\nabla B_{n,s}^{t-1}$ represents the gradient of loss 𝓛 with respect to $B_{n,s}$ at iteration t−1, and η is the learning rate. We use the ${𝓁}_{1}$-norm to evaluate the performance of bounding box regression. For each loss function (BIoU losses and CFIoU loss), the simulation experiment terminates when the iteration reaches T=200. The error curves in Figure 6b show that among 62,500 regression cases, the CFIoU loss is more effective than the BIoU losses in providing richer gradient information for the predicted box, leading to faster convergence of the loss between the predicted and target boxes, and allowing the predicted box to fit the target box completely (Algorithm 1).
**Algorithm 1** Simulation Experiment**Input:** ${\left\{{\left\{B_{n,s}\right\}}_{s=1}^{S}\right\}}_{n=1}^{N}$ denotes all the anchors at N=500 points, where S=5×5 is the number of combinations of different areas and aspect ratios. ${\left\{B_{i}^{gt}\right\}}_{i=1}^{5}$ is the set of target boxes that are fixed at (10, 10) with area 4, and have five aspect ratios. **Output:** Regression error E 1: (E,T)←(0,200) 2: Do bounding box regression: 3: for t=1 to T do 4:    for n=1 to N do 5:       for s=1 to S do 6:          if t≤0.8T then η=0.1 7:          else if t≤0.9T then η=0.01 8:          else η=0.001 9:          end if 10:        $\nabla B_{n,s}^{t-1}=\partial L\left(B_{n,s}^{t-1},B_{i}^{gt}\right)/\partial B_{n,s}^{t-1}$ 11:        $B_{n,s}^{t}=B_{n,s}^{t-1}+\eta \nabla B_{n,s}^{t-1}$ 12:        $E(t)=E(t)+\left|B_{n,s}^{t}-B_{i}^{gt}\right|$ 13:       end for 14:    end for 15: end for 16: Return E

### 4.3. Ablation Study

We performed an ablation study on the VisDrone2019-DET-test dataset using the YOLOv5s model to evaluate the advantages of different penalty terms in our proposed CFIoU loss function. Our results, shown in Table 1, demonstrate that the penalty term based on corner point distance performs better than the penalty term based on centroid distance (+1.1 Recall, +0.4 mAP@0.5, +0.1 mAP@0.5:0.95, (a) and (b)), and outperforms the IoU loss without any penalty term (+0.2 Recall, +0.1 mAP@0.5, +0.1 mAP@0.5:0.95, (b) and (c)). Additionally, the penalty term based on foreground information achieves superior performance over the IoU loss without any penalty term (+0.6 Recall, +0.6 mAP@0.5, +0.3 mAP@0.5:0.95, (c) and (d)). Finally, compared to the IoU loss with any penalty term, our proposed CFIoU loss function achieves the best performance (+1.9 Recall, +1.2 mAP@0.5, and +0.3 mAP@0.5:0.95).

### 4.4. Quantitative Results

Our experiment aims to verify the performance improvement effect of the CFIoU loss on object detection algorithms. To this end, we used advanced mainstream anchor-based YOLOv5 and anchor-free YOLOv8 object detection algorithms on two small object public datasets, VisDrone2019 and SODA-D, and compared the CFIoU loss with other BIoU losses. For the same dataset, YOLOv5 and YOLOv8 both used the same hyperparameter settings. Meanwhile, to demonstrate the superiority of our proposed loss function over traditional object detection algorithms, we conducted additional evaluation experiments using SSD, another popular one-stage method implemented in PyTorch.

#### 4.4.1. YOLOv5 and YOLOv8 on VisDrone2019

The VisDrone2019 dataset is an important benchmark dataset for studying unmanned aerial vehicle (UAV) object detection algorithms, contains 10 categories of objects, and is particularly suitable for small object detection tasks. In this dataset, we used the YOLOv5 object detection algorithm trained with the CFIoU loss and compared it with the BIoU (IoU, GIoU, DIoU, CIoU, and EIoU) loss functions. The training set consists of VisDrone2019-DET-train and VisDrone2019-DET-val, totaling 7019 images. The test set is the VisDrone2019-DET-test-dev dataset, which contains 1610 images.

**Figure 7:**
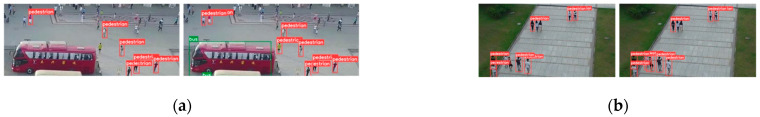
Detection examples using YOLOv5s trained on the VisDrone2019 dataset. Visualization samples are chosen from VisDrone2019-DET-test-challenge. (**a**,**b**): Left: ${𝓛}_{EIoU}$, right: ${𝓛}_{CFIoU}$.

**Figure 8:**
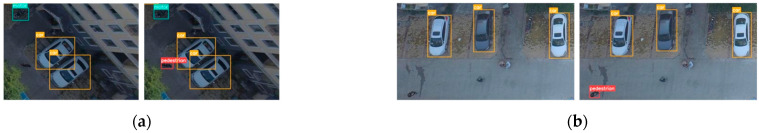
Detection examples using YOLOv5s trained on the VisDrone2019 dataset. Visualization samples are chosen from VisDrone2019-DET-test-challenge. (**a**,**b**): Left: ${𝓛}_{IoU}$, right: ${𝓛}_{CFIoU}$.

According to the results in Table 2, the CFIoU loss plays an important role in improving the performance of both anchor-based YOLOv5s and anchor-free YOLOv8s object detection algorithms, demonstrating the strong compatibility of this loss function. In the test results, the YOLOv5s model with the integrated CFIoU loss showed the largest improvement, with a 3.12% increase in recall rate, a 2.73% increase in mAP@0.5, and a 1.91% increase in mAP@0.5:0.95. Meanwhile, compared to the baseline model, the YOLOv5s model with integrated GIoU, DIoU, and CIoU losses experienced a performance decline, largely due to the degradation of these loss functions during training. However, the EIoU loss, which splits the aspect ratio on top of the CIoU loss, did bring improvement, with a 1.56% increase in recall rate, a 2.05% increase in mAP@0.5, and a 1.91% increase in mAP@0.5:0.95. Compared to the EIoU loss, the CFIoU loss solved the degradation problem of BIoU losses and placed greater emphasis on utilizing target foreground information, resulting in a higher performance improvement. It is noteworthy that the YOLOv8s model with the integrated CFIoU loss achieved the best performance on the same metrics. We used YOLOv5 trained on the VisDrone2019 dataset to detect a subset of examples from the VisDrone2019-DET-test-challenge. We compared the EIoU loss, which performed better among the BIoU losses, with our proposed CFIoU loss. As shown in Figure 7, the model fused with the CFIoU loss performed better at finding targets than the model fused with the EIoU loss. Additionally, we compared the IoU loss with the CFIoU loss and presented some examples in Figure 8. From the inference results, it can be seen that the YOLOv5 model fused with the CFIoU loss has a significant effect in capturing small targets.

#### 4.4.2. YOLOv5 and YOLOv8 on SODA-D

The SODA-D dataset exhibits rich diversity in terms of location, weather, period, scene, and shooting angle. Moreover, SODA-D boasts very high-resolution and high-quality images and contains nine categories of objects, making it ideal for small object detection tasks. The training set contains 12,383 images, the validation set contains 5017 images, and the test set contains 7528 images. It should be noted that the training and testing of YOLOv5s and YOLOv8s on the SODA-D dataset are consistent with those on the VisDrone2019 dataset. As shown in Table 3, the performance of YOLOv5s and YOLOv8s with the fusion of CFIoU loss on the SODA-D test set is the best and significantly improved, which is consistent with the results on the VisDrone2019 dataset. This fully demonstrates that the CFIoU loss has superior and effective performance compared to BIoU losses.

As shown in Table 3, consistent with the performance on the VisDrone2019 dataset, YOLOv5s and YOLOv8s fused with the CFIoU loss achieve the best results on the SODA-D test set with a significant performance improvement. The consistent performance on both the VisDrone2019 and SODA-D datasets demonstrates the superiority and effectiveness of the CFIoU loss over the BIoU losses.

#### 4.4.3. SSD on VisDrone2019

Both YOLOv5 and YOLOv8 exhibit excellent performance in detecting small objects, and the proposed CFIoU loss function can further enhance their detection capabilities. To demonstrate the superiority of our proposed loss function over traditional object detection algorithms, we conducted additional evaluation experiments using SSD, another popular one-stage method implemented in PyTorch. We adopted the same training and testing setup as YOLOv5 and YOLOv8 on the VisDrone2019 dataset and set the iteration number to 32,400 based on experimental results and data fitting performance. Performance metrics for each type of loss were reported in Table 4, with AP (the average of 10 mAPs at different IoU thresholds, mAP@0.5:0.95) = (AP50 + AP55 + … + AP95)/10 and AP75 (mAP@0.75) used as evaluation metrics.

It is evident that the SSD object detection algorithm does not perform well in detecting small objects. As shown in Table 4, SSD models trained with GIoU, DIoU, CIoU, and EIoU losses all showed performance degradation in AP metrics compared to the baseline model. In addition, except for a slight increase with GIoU loss, all other losses resulted in decreased performance in AP75 metrics. This indicates that BIoU losses perform poorly in algorithms that are not good at detecting small objects, such as SSD. However, the SSD algorithm with CFIoU loss showed a remarkable improvement of 5.59% and 5.37% in AP and AP75 metrics, respectively, demonstrating the advantage of CFIoU loss in small object detection. This also indicates that even for object detection algorithms with poor performance in detecting small objects, the use of CFIoU loss in training can still lead to improved small object detection performance.

### 4.5. Discussion

The results from Table 2 and Table 3 demonstrate that the CFIoU loss is compatible with both anchor-based YOLOv5 and anchor-free YOLOv8, leading to improved model training and performance. However, the CFIoU loss is more adaptable to anchor-based object detection algorithms than anchor-free algorithms in terms of performance improvement. This is because, in anchor-based object detection algorithms, the predefined prior boxes facilitate the accurate matching of the size and shape of target objects and guide bounding box regression using the IoU loss function. Moreover, the information from prior boxes can be utilized to constrain the regression range, reducing the difficulty of regression. On the other hand, anchor-free object detection algorithms lack prior box information and require determining the position and size of the true bounding boxes using other means to guide regression. Although this approach is more flexible, it is also more challenging due to the absence of constraints on the regression range, making it difficult to achieve the same level of performance as anchor-based algorithms. Nevertheless, the CFIoU loss remains effective for anchor-free algorithms.

### 4.6. Further Work

CFIoU is a variant of IoU. In the original Non-Maximum Suppression (NMS), IoU is used to suppress redundant detection boxes, where the overlapping area is the only factor considered. This often leads to erroneous suppression in the presence of occlusion. In our future work, we will further investigate CFIoU-NMS, which takes into account the distance between the four corners of the two boxes in CFIoU as well as the foreground information of the target box in the suppression criterion. This may bring significant benefits to dense object detection or object detection in the presence of occlusion.

## 5. Conclusions

In this paper, we propose a novel loss function, namely, the CFIoU loss, to guide bounding box regression. We address the issue of BIoU loss degradation to IoU loss during model training by designing a penalty term that normalizes the distance between corresponding corner points of the bounding boxes. Additionally, we introduce a foreground-target-based penalty term to better emphasize the spatial information between the predicted and ground truth bounding boxes. This penalty gives a larger punishment to predicted bounding boxes with poor regression performance, facilitating better fitting to the ground truth bounding boxes. Finally, we demonstrate the superior performance of the CFIoU loss in both anchor-based and anchor-free object detection algorithms, as well as its effectiveness in improving small object detection through extensive experiments.

## Figures and Tables

**Table 1:** Ablation study of each loss term.

Loss	IoU	Centroids	Corner Points	Foreground Areas	Recall	mAP@0.5	mAP@0.5:0.95
(a)	√	√			31.2	28.9	15.7
(b)	√		√		**32.3 (↑3.53%)**	**29.3 (↑1.38%)**	**15.8 (↑0.64%)**
(c)	√				32.1	29.2	15.7
(d)	√			√	32.7	29.8	16.0
(e)	√		√	√	**33.1**	**30.1**	**16.0**

**Table 2:** Quantitative comparison of YOLOv5s and YOLOv8s trained using ${𝓛}_{IoU}\left(\mathrm{baseline}\right)$, ${𝓛}_{GIoU}$, ${𝓛}_{DIoU}$, ${𝓛}_{CIoU}$, ${𝓛}_{EIoU}$, and ${𝓛}_{CFIoU}$, respectively. The reported results are on the test set of VisDrone2019.

Loss/Evaluation	YOLOv5s			YOLOv8s		
	Recall	mAP@0.5	mAP@0.5:0.95	Recall	mAP@0.5	mAP@0.5:0.95
${𝓛}_{IoU}$	32.1	29.3	15.7	34.9	33.4	19.3
${𝓛}_{GIoU}$	31.7	29.1	15.7	34.1	32.5	18.7
R I.% ^1^	−1.25%	−0.68%	-	−2.29%	−2.69%	−3.11%
${𝓛}_{DIoU}$	31.2	28.9	15.7	35.2	33.5	19.1
R I.% ^1^	−2.80%	−1.37%	-	**+0.86%**	**+0.30%**	−1.04%
${𝓛}_{CIoU}$	31.5	29.2	15.7	35.0	33.2	19.0
R I.% ^1^	−1.87%	−0.34%	-	−0.29%	−0.60%	−1.55%
${𝓛}_{EIoU}$	32.6	29.9	16.0	35.2	33.1	19.0
R I.% ^1^	**+1.56%**	**+2.05%**	**+1.91%**	**+0.86%**	−0.90%	−1.55%
${𝓛}_{CFIoU}$	**33.1**	**30.1**	**16.0**	**35.5**	**33.6**	**19.3**
R I.% ^1^	**+3.12%**	**+2.73%**	**+1.91%**	**+1.72%**	**+0.60%**	-

^1^ R I.% = Relative improv. %.

**Table 3:** Quantitative comparison of YOLOv5s and YOLOv8s trained using ${𝓛}_{IoU}\left(\mathrm{baseline}\right)$, ${𝓛}_{GIoU}$, ${𝓛}_{DIoU}$, ${𝓛}_{CIoU}$, ${𝓛}_{EIoU}$, and ${𝓛}_{CFIoU}$, respectively. The reported results are on the test set of SODA-D.

Loss/ Evaluation	YOLOv5s			YOLOv8s		
	Recall	mAP@0.5	mAP@0.5:0.95	Recall	mAP@0.5	mAP@0.5:0.95
${𝓛}_{IoU}$	15.00	10.70	3.64	11.90	8.46	3.21
${𝓛}_{GIoU}$	13.10	9.56	3.33	11.80	8.48	3.27
R I.% ^1^	−12.67%	−10.65%	−8.52%	−8.40%	**+0.24%**	**+1.87%**
${𝓛}_{DIoU}$	13.80	10.00	3.40	11.90	8.53	3.24
R I.% ^1^	−8.00%	−6.54%	−6.59%	-	**+0.83%**	**+0.93%**
${𝓛}_{CIoU}$	14.10	10.30	3.33	11.80	8.52	3.25
R I.% ^1^	−6.00%	−3.74%	−8.52%	−8.40%	**+0.71%**	**+1.25%**
${𝓛}_{EIoU}$	15.6	11.6	3.91	12.1	8.62	3.29
R I.% ^1^	**+4.00%**	**+8.41%**	**+7.42%**	**+1.68%**	**+1.89%**	**+2.49%**
${𝓛}_{CFIoU}$	**15.9**	**12.1**	**4.16**	**12.3**	**8.77**	**3.34**
R I.% ^1^	**+6.00%**	**+13.08%**	**+14.29%**	**+3.36%**	**+3.66%**	**+4.05%**

^1^ R I.% = Relative improv. %.

**Table 4:** Quantitative comparison of SSD trained using ${𝓛}_{IoU}\left(\mathrm{baseline}\right)$, ${𝓛}_{GIoU}$, ${𝓛}_{DIoU}$, ${𝓛}_{CIoU}$, ${𝓛}_{EIoU}$, and ${𝓛}_{CFIoU}$, respectively. The reported results are on the test set of VisDrone2019.

Loss/Evaluation	AP	AP75
${𝓛}_{IoU}(\mathrm{baseline})$	7.382	7.453
${𝓛}_{GIoU}$	7.247	**7.519**
Relative improv. %	−1.83%	**+0.89%**
${𝓛}_{DIoU}$	7.214	7.331
Relative improv. %	−2.28%	−1.64%
${𝓛}_{CIoU}$	7.155	7.095
Relative improv. %	−3.08%	−4.80%
${𝓛}_{EIoU}$	7.340	7.037
Relative improv. %	−0.57%	−5.58%
${𝓛}_{CFIoU}$	**7.795**	**7.853**
Relative improv. %	**+5.59%**	**+5.37%**

## Data Availability

The data that support the findings of this study are available from the corresponding author upon reasonable request.
